# Supplementary material for: Predictors of students’ participation in a learning environment survey with annual follow-ups
Source: PLoS One. 2021 Jun 25;16(6):e0253773. doi: 10.1371/journal.pone.0253773 (PMC8232400; doi:10.1371/journal.pone.0253773)
Supplement: Attachment — Submitted filename: PONE-D-21-11288_reviewer.pdf [file pone.0253773.s001.pdf]

PLOS ONE

Predictors of students’ participation in a learning environment survey with annual follow-ups

--Manuscript Draft--

**Manuscript Number:**	PONE-D-21-11288
**Article Type:**	Research Article
**Full Title:**	Predictors of students’ participation in a learning environment survey with annual follow-ups
**Short Title:**	Predictors of students’ survey participation
**Corresponding Author:**	Tore Bonsaksen<br>Hogskolen i Innlandet Fakultet for helse- og sosialvitenskap<br>Elverum, NORWAY
**Keywords:**	academic performance; approaches to studying; attrition; exam grades; learning environment; research participation,
**Abstract:**	Background<br><br>Longitudinal research is one effective way to gauge changes in a student cohort over time, however attrition in these studies is typically high, which can result in study bias. This study explored learning environment factors, approaches to studying, and academic performance as predictors of occupational therapy students’ consistent participation in data collection conducted over three years of their professional program.<br><br>Method<br><br>A longitudinal study of Norwegian occupational therapy students (analyzed n = 240) was conducted. Logistic regression analysis was used to explore occupational therapy students’ perceptions of the learning environment, their approaches to studying, and exam grades as they related to the likelihood of consistent participation at three annual surveys.<br><br>Results<br><br>Annual response rates varied between 55.1%, and 65.6%, and consistent participation was observed among 49.2%. The fully adjusted regression models showed that higher strategic approach scores increased the odds of consistent participation (adjusted OR: 1.04, $p < 0.01$), whereas higher surface approach scores decreased the odds of consistent participation (adjusted OR: 0.95, $p < 0.05$). Neither sociodemographic factors, learning environment factors nor academic performance predicted participation over time.<br><br>Conclusions<br><br>Researchers can anticipate relatively high levels of attrition in longitudinal studies of occupational therapy students, but attrition seems to be largely proportional between groups. However, completers in longitudinal studies may be somewhat more well-organized and academically oriented than drop-outs.
**Order of Authors:**	Elaina DaLomba
	Astrid Gramstad
	Susanne G. Johnson
	Tove Carstensen
	Linda Stigen
	Gry Mørk

Powered by Editorial Manager® and ProduXion Manager® from Aries Systems Corporation

	Trine A. Magne
	Tore Bonsaksen
**Additional Information:**	
**Question**	**Response**
**Financial Disclosure**<br><br>Enter a financial disclosure statement that describes the sources of funding for the work included in this submission. Review the submission guidelines for detailed requirements. View published research articles from *PLOS ONE* for specific examples.<br><br>This statement is required for submission and **will appear in the published article** if the submission is accepted. Please make sure it is accurate.<br><br>**Unfunded studies**<br>Enter: *The author(s) received no specific funding for this work.*<br><br>**Funded studies**<br>Enter a statement with the following details:<br>• Initials of the authors who received each award<br>• Grant numbers awarded to each author<br>• The full name of each funder<br>• URL of each funder website<br>• Did the sponsors or funders play any role in the study design, data collection and analysis, decision to publish, or preparation of the manuscript?<br>• **NO** - Include this sentence at the end of your statement: *The funders had no role in study design, data collection and analysis, decision to publish, or preparation of the manuscript.*<br>• **YES** - Specify the role(s) played.<br><br>* typeset	The authors received no specific funding for this work.
**Competing Interests**<br><br>Use the instructions below to enter a competing interest statement for this submission. On behalf of all authors, disclose any competing interests that could be perceived to bias this work—acknowledging all financial support and any other relevant financial or non-	The authors have declared that no competing interests exist.

financial competing interests.<br><br>This statement is required for submission and **will appear in the published article** if the submission is accepted. Please make sure it is accurate and that any funding sources listed in your Funding Information later in the submission form are also declared in your Financial Disclosure statement.<br><br>View published research articles from *PLOS ONE* for specific examples.<br><br>**NO authors have competing interests**<br><br>Enter: *The authors have declared that no competing interests exist.*<br><br>**Authors with competing interests**<br><br>Enter competing interest details beginning with this statement:<br><br>*I have read the journal's policy and the authors of this manuscript have the following competing interests: [insert competing interests here]*<br><br>* typeset	
**Ethics Statement**<br><br>Enter an ethics statement for this submission. This statement is required if the study involved:<br><br>• Human participants<br>• Human specimens or tissue<br>• Vertebrate animals or cephalopods<br>• Vertebrate embryos or tissues<br>• Field research<br><br>Write "N/A" if the submission does not require an ethics statement.<br><br>General guidance is provided below.<br>Consult the submission guidelines for	Approval for collecting, storing and utilizing the de-identified data was granted on October 12, 2017 by the Norwegian Center for Research Data (project no. 55875). Written informed consent was provided from all participants.

detailed instructions. **Make sure that all information entered here is included in the Methods section of the manuscript.**<br><br>**Format for specific study types**<br><br>**Human Subject Research (involving human participants and/or tissue)**<br>• Give the name of the institutional review board or ethics committee that approved the study<br>• Include the approval number and/or a statement indicating approval of this research<br>• Indicate the form of consent obtained (written/oral) or the reason that consent was not obtained (e.g. the data were analyzed anonymously)<br><br>**Animal Research (involving vertebrate animals, embryos or tissues)**<br>• Provide the name of the Institutional Animal Care and Use Committee (IACUC) or other relevant ethics board that reviewed the study protocol, and indicate whether they approved this research or granted a formal waiver of ethical approval<br>• Include an approval number if one was obtained<br>• If the study involved *non-human primates*, add *additional details* about animal welfare and steps taken to ameliorate suffering<br>• If anesthesia, euthanasia, or any kind of animal sacrifice is part of the study, include briefly which substances and/or methods were applied<br><br>**Field Research**<br><br>Include the following details if this study involves the collection of plant, animal, or other materials from a natural setting:<br>• Field permit number<br>• Name of the institution or relevant body that granted permission	
**Data Availability**<br><br>Authors are required to make all data underlying the findings described fully available, without restriction, and from the time of publication. PLOS allows rare	Yes - all data are fully available without restriction

Powered by Editorial Manager® and ProduXion Manager® from Aries Systems Corporation

exceptions to address legal and ethical concerns. See the PLOS Data Policy and FAQ for detailed information.<br><br>A Data Availability Statement describing where the data can be found is required at submission. Your answers to this question constitute the Data Availability Statement and **will be published in the article**, if accepted.<br><br>**Important:** Stating 'data available on request from the author' is not sufficient. If your data are only available upon request, select 'No' for the first question and explain your exceptional situation in the text box.<br><br>Do the authors confirm that all data underlying the findings described in their manuscript are fully available without restriction?	
**Describe where the data may be found in full sentences. If you are copying our sample text, replace any instances of XXX with the appropriate details.**<br><br>• If the data are **held or will be held in a public repository**, include URLs, accession numbers or DOIs. If this information will only be available after acceptance, indicate this by ticking the box below. For example: *All XXX files are available from the XXX database (accession number(s) XXX, XXX.*).<br>• If the data are all contained **within the manuscript and/or Supporting Information files**, enter the following: *All relevant data are within the manuscript and its Supporting Information files.*<br>• If neither of these applies but you are able to provide **details of access elsewhere**, with or without limitations, please do so. For example:<br><br>*Data cannot be shared publicly because of [XXX]. Data are available from the XXX Institutional Data Access / Ethics Committee (contact via XXX) for researchers who meet the criteria for*	The data underlying the results presented in the study are available from Inland Norway University of Applied Sciences on request to the project manager after completion of the study. E-mail tore.bonsaksen@inn.no

access to confidential data.<br><br>*The data underlying the results presented in the study are available from (include the name of the third party and contact information or URL).*<br>• This text is appropriate if the data are owned by a third party and authors do not have permission to share the data.<br><br>* typeset	
Additional data availability information:	Tick here if the URLs/accession numbers/DOIs will be available only after acceptance of the manuscript for publication so that we can ensure their inclusion before publication.

manuscript Click here to access/download;Manuscript;Predictors of students participation (PLOS ONE submission).docx

Predictors of students' participation in a learning environment survey with annual follow-ups

Elaina DaLomba [1], Astrid Gramstad [2, 3], Susanne Grødem Johnson [4], Tove Carstensen [5], Linda Stigen [6], Gry Mørk [7], Trine Magne [5], & Tore Bonsaksen [7, 8, *]

[1] Occupational Therapy Department, Samuel Merritt University, Oakland, CA, United States of America

[2] Department of health and care sciences, Faculty of health science, UiT – the arctic university of Norway

[3] Centre for care research, north

[4] Department of Health and Social Functioning, Western Norway University of Applied Sciences, Bergen, Norway

[5] Department of Neuroscience and Movement science, Norwegian University of Science and Technology (NTNU), Trondheim, Norway

[6] Department of Health Sciences, Norwegian University of Science and Technology (NTNU), Gjøvik, Norway

[7] Faculty of Health Studies, VID Specialized University, Sandnes, Norway

[8] Department of Health and Nursing Sciences, Faculty of Social and Health Sciences, Inland Norway University of Applied Sciences, Elverum, Norway

*Corresponding author: tore.bonsaksen@inn.no (TB)

Abstract

Background: Longitudinal research is one effective way to gauge changes in a student cohort over time, however attrition in these studies is typically high, which can result in study bias. This study explored learning environment factors, approaches to studying, and academic performance as predictors of occupational therapy students' consistent participation in data collection conducted over three years of their professional program.

Method: A longitudinal study of Norwegian occupational therapy students (analyzed $n = 240$) was conducted. Logistic regression analysis was used to explore occupational therapy students' perceptions of the learning environment, their approaches to studying, and exam grades as they related to the likelihood of consistent participation at three annual surveys.

Results: Annual response rates varied between 55.1 %, and 65.6 %, and consistent participation was observed among 49.2 %. The fully adjusted regression models showed that higher strategic approach scores increased the odds of consistent participation (adjusted OR: 1.04, $p < 0.01$), whereas higher surface approach scores decreased the odds of consistent participation (adjusted OR: 0.95, $p < 0.05$). Neither sociodemographic factors, learning environment factors nor academic performance predicted participation over time.

Conclusions: Researchers can anticipate relatively high levels of attrition in longitudinal studies of occupational therapy students, but attrition seems to be largely proportional between groups. However, completers in longitudinal studies may be somewhat more well-organized and academically oriented than drop-outs.

Keywords: academic performance, approaches to studying, attrition, exam grades, learning environment, research participation,

Introduction

Student participation in learning environments has become an issue of increasing concern for universities. Often referred to as engagement, evidence shows that active involvement in learning activities correlates with academic retention and success [1], thus understanding what leads to engagement is inherently meaningful to educators. Student engagement is a broad construct, but appears to be multi-factorial, having cognitive, emotional, and behavioral facets, and multi-directional impacts [2]. For example, studies have shown learner characteristics that lead to engagement, such as self-regulation and persistence [3], those who engage in self-reflection and self-assessment [4], and the use of deep and strategic study approaches [5], correlated with increased academic success. Conversely, studies examining learning environments show that clear structure, emotional and academic support, relationships with peers and instructors, opportunities for independent decision-making [2, 6], clear meaning and relevance to goals [7], and quality of teaching and manageability of workload [8] can impact engagement. Increasing student engagement is a target of many institutions, as many of the studies mentioned above show that engagement, and thus academic success, is malleable [3, 4, 8].

Student approaches to studying and learning have been explored in some depth. Originating in Martin and Saljo's [9] (1976) work and expanded by Entwistle [10], study behaviors are characterized as *deep*, such as analyzing and relating ideas through critical thinking, and *surface,* such as rote learning and memorization in which broader connections are not made nor deeper meaning sought [11]. With further exploration, a third *strategic* approach that addresses student organization and self-regulation around work was added [12]. Recent evidence from occupational therapy student studies showed that students who adopt deep and strategic approaches tend to have higher reported self-efficacy and positive mental

health [13], and are more inclined to adopt a positive outlook on the broader learning outcomes of the study program.

Longitudinal research follows a particular group of individuals over time, allowing researchers to capture changes, and amount of change, with exposure to different variables [14]. Longitudinal studies can be effectively conducted in higher education because most students are present over a period of semesters and years. However, maintaining participant engagement in research and reducing participant attrition (dropouts) are crucial for the value and generalizability of data obtained [15]. Some attrition is to be expected in longitudinal studies and may be due to loss of interest or lack of appreciation of the relevance of their individual situation to the phenomena being studied [16]. Repeated data collection may be experienced as burdensome to some participants, which can lead to incomplete responses and attrition [16]. Teague and colleagues [17] noted that making participation as easy and convenient as possible, “barrier reduction” such as providing reminders and keeping items to a minimum, was most impactful in increasing retention.

Longitudinal research is used frequently to study college student behaviors, characteristics, as well as to evaluate the development of students’ learning. The purpose of such studies can be to identify program needs or to confirm and support use of existing methods. However, as with many studies, results and their meaning are often impacted by attrition. For example, in their study of occupational therapy students in team-based learning, [18] found that the retention of students from the first to the second time of measurement was limited to a degree that is decreased the generalizability of the results. Similarly, in their study of emotional intelligence in diverse student groups, Gribble, Ladyshewsky, and Parsons [19] noted that their high attrition rate (50%), particularly in the control group, could have led to erroneous comparisons between the groups.

To the best of our knowledge, there is no research that addresses the impact of specific learner, or learning environment characteristics, on the likelihood of student participation and retention in longitudinal studies. However, when considering conducting intervention studies in higher education, it is crucial to have knowledge of these characteristics, since the recruitment and retention of participants can increase the validity and generalizability of them [20]. Moreover, if dropouts from longitudinal studies are systematically different from completers, this will decrease the validity of the results obtained from such studies. It seems possible that student background factors, their academic abilities, and their perceptions of the learning environment and approaches to studying may differ between those who are interested and willing to participate in longitudinal research in higher education and those who are not. Therefore, more knowledge about factors predictive of retention and dropout from longitudinal studies of higher education students can give some indication of the external validity of such studies and the biases they may have.

The aim of the longitudinal study was to examine (i) the rates of student participation in the research study across time, and (ii) learning environment factors, approaches to studying, and academic performance as predictors of occupational therapy students' consistent participation in the three waves of data collection conducted during the course of the study program.

Methods

Design and study context

The study is based on a longitudinal study of Norwegian occupational therapy students' perceptions of the learning environment and approaches to studying. All occupational therapy education programs in Norway are three-year undergraduate programs. In the current study,

the students' participation in each year of study was examined, and predictors of consistent participation (participation in the three years of study) were explored.

Procedure and eligible participants

About midway through each of the three study years (i.e., in the December and January months), occupational therapy students at six higher education institutions in Norway were approached for possible participation in the study. The purpose and design of the study was outlined in classroom session at each of the education institutions, followed by an invitation to participate. The questionnaires were completed within the same session, or (in a few cases) later at a time and place of the students' convenience. From the six education programs, 305 students were eligible participants.

Measurement

Sociodemographic variables

Age (in years) was registered as a continuous variable. Gender (male = 0, female = 1), having prior experience from higher education (no = 0, yes = 1) and having occupational therapy as the highest prioritized line of education at the time of enrolment (no = 0, yes = 1) were registered as categorical variables.

The learning environment

The original *Course Experience Questionnaire (CEQ)* [21] consists of 30 items distributed onto five scales: clear goals and standards, emphasis on independence, good teaching, appropriate workload, and appropriate assessment. In addition to the 30 items, one item assesses the students' general satisfaction with the course. The 37 items long version of the CEQ includes a sixth scale; generic skills [22-24]. The validated Norwegian translation of the long version [25] was used in the present study. Higher scores on the scales indicate that the respondent perceives the course to have (1) clearly established and disseminated goals; (2)

high levels of student autonomy and independence; (3) teaching that engages and involves the students; (4) a workload that is not too high; (5) assessment forms that promote and support learning; and the course is felt to (6) support the transfer of content knowledge and skills to the relevant work context. Among the participants in the first wave (first-year students), internal consistency of the scales was 0.73 (clear goals and standards), 0.63 (emphasis on independence), 0.70 (good teaching), 0.69 (appropriate workload), 0.45 (appropriate assessment), and 0.83 (generic skills) [26]. In view of the internal consistency results, subsequent analyses of the 'appropriate assessment' scale were not pursued.

Approaches to studying

Study approaches were measured with the *Approaches and Study Skills Inventory for Students* [27]and the students used a previously validated Norwegian translation of the instrument [28]. The ASSIST consists of 52 statements to which the respondent is asked to rate his or her level of agreement (1 = disagree, 2 = disagree somewhat, 3 = unsure, 4 = agree somewhat, 5 = agree). The instrument has a three-factor structure, a structure recently replicated in a cross-cultural study of undergraduate occupational therapy students [26]. The items are organized accordingly into three main scales (the *deep, strategic,* and *surface* approaches to studying). Scale scores are calculated by adding the scores on the relevant items. Among the participants in the first wave (first-year students), internal consistency estimates (Cronbach's α) for the study approach scales were 0.71 (deep approach), 0.84 (strategic approach), and 0.76 (surface approach) [29].

Academic performance

As the education institutions used a different number of exams in each study year, the students' average exam performance (based on the completed number of exams) was first calculated within each study year, and then across the whole study period. The students' average exam grade scores were based on the qualitative descriptors related to the students'

exam grades: fail = 1, sufficient = 2, satisfactory = 3, good = 4, very good = 5, and excellent = 6 [30].

Data analysis

The sample was described with descriptive statistics; i.e., means and standard deviations for continuous variables and frequencies and percentages for categorical variables. Scores on continuous measures, such as grades and the learning environment and study approach scales, were averaged across the number of measurement occasions on which the students had participated. Consistent participation in the study was defined as having completed and returned the questionnaires on all three measurement occasions. Single and multiple binary logistic regression analyses were performed using consistent participation as the outcome variable. The independent variables included in the single logistic regression analyses were age, gender, educational priority, and prior higher education (representing the background variables); scores on clear goals and standards, student autonomy, good teaching, appropriate workload, and generic skills (representing the learning environment variables); scores on deep approach, strategic approach and surface approach scales (representing the approaches to studying); and average exam grade across all study years. In the adjusted (multiple) logistic regression analysis, background variables were included only if they had a statistically significant bivariate association with the outcome. Grades, all learning environment scales, and study approach scales were included in the adjusted analysis, regardless of their unadjusted association with the outcome. Effect sizes were reported as odds ratio (OR) with corresponding 95 % confidence interval (95 % CI).

Research ethics

Approval for collecting, storing and utilizing the de-identified data was granted on October 12, 2017 by the Norwegian Center for Research Data (project no. 55875).

Results

Response rates

Of the 305 eligible students, 187 students participated in the first study year (response rate 61.3 %). In the second year of study, 168 students participated, representing a response rate of 55.1 %. In the third year of study, 200 students participated, representing a response rate of 65.6 % and an increase in response rate compared to both previous years.

A total of 263 students (86.2 %) participated at least once during the three-year study period. We were unable to track the exam grades of 23 students, and these were removed from the analyses. Thus, 240 students constituted the final study sample. Of these, 118 students (49.2 %) participated consistently at all three measurement occasions.

Participant characteristics

The sample mean age was 22.6 years, and 190 of the 240 participants (79.2 %) were women. At enrollment, occupational therapy was the highest priority line of education for 63 % of the students, while 41 % had previous experience from university level studying. Table 1 displays the students' background characteristics, averaged grade and averaged perceptions of the learning environment and approaches to studying.

Table 1. Sample characteristics (*n* = 240)

Variables	Scale range	Values
Sociodemographic variables		*M (SD)*
Age (years)		22.6 (4.4)
		n (%)
Female gender		190 (79.2)
Occupational therapy was priority line of study		152 (63.3)
With prior higher education experience		98 (40.8)
Learning environment		*M (SD)*
Clear goals and standards	5-25	16.8 (3.2)
Student autonomy	6-30	18.3 (3.5)
Good teaching	8-40	26.3 (5.1)
Appropriate workload	5-25	15.2 (3.3)
Generic skills	6-30	23.8 (3.6)
Satisfaction with the study program	1-5	3.8 (0.8)
Approaches to studying		*M (SD)*
Deep approach	16-80	57.1 (8.0)
Strategic approach	20-100	71.8 (8.8)
Surface approach	16-80	46.3 (8.5)
Academic performance		
Average exam grade	1-6	4.0 (0.8)

Note. Scale range is the possible scale range. Satisfaction with the study program is one item.

Predictors of consistent participation in the research study

Table 2 displays the results from the unadjusted regression analysis (left side) and the adjusted regression analysis (right side), using consistent participation as the dependent variable. None of the sociodemographic variables or the learning environment variables significantly predicted change in the odds for consistent participation. However, each unit increase in strategic approach score increased the odds of consistent participation, even in the fully adjusted model (adjusted OR: 1.04, $p < 0.01$). Conversely, each unit increase in surface approach score decreased the odds of consistent participation, even in the fully adjusted model (adjusted OR: 0.95, $p < 0.05$). Higher grades were associated with higher odds of consistent study participation in the unadjusted model, but the association was no longer statistically significant in the adjusted model.

Table 2. Single and multiple binary logistic regression analyses showing associations with consistent participation in the research study

Independent variables	Unadjusted associations			Adjusted associations		
Sociodemographic variables	OR	95 % CI	*p*	OR	95 % CI	*p*
Age	1.00	0.94-1.06	0.94			
Gender	0.87	0.47-1.62	0.87			
Priority line of study	1.15	0.68-1.95	0.60			
Prior higher education	0.74	0.44-1.24	0.25			
Learning environment						
Clear goals and standards	1.05	0.97-1.14	0.24	1.01	0.91-1.13	0.81
Student autonomy	1.02	0.95-1.10	0.61	1.03	0.93-1.13	0.60
Good teaching	1.00	0.95-1.05	0.91	0.99	0.92-1.07	0.85
Appropriate workload	1.03	0.95-1.11	0.50	0.97	0.88-1.07	0.56
Generic skills	0.99	0.92-1.06	0.75	0.93	0.83-1.03	0.15
Satisfaction with study program	1.11	0.82-1.52	0.50	0.99	0.63-1.55	0.96
Approaches to studying						
Deep approach	1.00	0.97-1.03	0.97	1.00	0.96-1.03	0.86
Strategic approach	1.04	1.01-1.07	< 0.05	1.04	1.01-1.08	< 0.05
Surface approach	0.96	0.93-0.99	< 0.01	0.95	0.92-0.99	< 0.05
Academic performance						

Mean exam grade	1.48	1.05-2.09	< 0.05	1.25	0.86-1.82	0.24

Note. In the adjusted model, all variables are entered into the equation in one block. Parameters for the adjusted model: Model $\chi^2 = 19.6$, $p < 0.05$. Nagelkerke $R^2 = 0.11$, Cox-Snell $R^2 = 0.08$.

Discussion

The aim of the study was to examine learning environment factors and approaches to studying as predictors of occupational therapy students' consistent participation in three waves of data collection in a learning environment survey. The students' scores on the strategic and surface approach scales were significantly associated with higher and lower odds for consistent participation in the research study, respectively. None of the background variables, learning environment variables, or academic performance significantly predicted consistent participation in the study.

Response rates across time

In this study, student response rates varied at each point of measurement, but were modest overall. This is consistent with the findings of others who studied allied health students longitudinally [18, 19, 31, 32]. Comparisons beyond this are difficult, as many studies do not report the point in time at which participants dropped out if capturing data at multiple points in time, or reasons for non-participation/drop-out, offering only overall outcomes. Of course, participants have a right to discontinue their involvement in research studies, thus trying to ascertain reasons for dropping out may not be appropriate. Even the term ”response rate” is defined and derived in a variety of ways, often not clearly described in studies [33], further decreasing the effectiveness and potential value of comparisons between studies. Our study found the lowest level of participation in the second study year, which may represent some unknown factor about the intensity of the workload at that time in the program. Conversely, the highest participation rate was shown in the third study year possibly indicating stronger inclinations towards contributing to the knowledge base in a profession they are soon to enter. Moreover, at the time of the data collection in the third year, the students were preparing for their bachelor's thesis, which involves planning and conducting a small-scale research project. Thus, the students' involvement in thesis work at the time may also have served to

increase their interest in and willingness to participate in the study in the third year. All of these connections, however, would require further investigation. Nonetheless, this study provides novel information of occupational therapy students' participation rates over three years of data collection, and these appear to be consistent with longitudinal study norms [17].

There is some evidence that intervention, versus observational studies, may show higher retention rates in allied health students. For example, DaLomba et al. [34] studied the impacts of an embedded librarian on information literacy skills (ILS) of occupational therapy students and reported a 92% retention rate. It is plausible that the novel addition of the librarian's presence in the course, providing direct teaching, mentoring, and consistent communication of her purpose (to enhance ILS), acted as a prompt to students to complete study protocols at both collection times (beginning and end of semester). The high participation rate in the study may also be attributed to study goals being highly related to course goals, thus increasing relevance and meaning of study participation for the students. To understand the purpose and relevance of course work correlates with increased engagement academically [7, 35, 36] therefore it seems reasonable that student engagement in study procedures could have been enhanced by these factors.

Predictors of consistent participation in the research study

This study found that only two features of student approaches to studying and learning impacted persistent participation. Students who had higher scores on strategic learning were more likely, whereas students who had higher scores on surface learning were less likely to participate in all of the three data collection procedures. Since strategic learners are those that actively observe the dynamics of their environment and seek ways to meet the standards of those who assess their learning [10] it may be that these students responded to requests to participate more frequently due to a perceived benefit to doing so, such as enhancing chances of getting better grades in the course. Alternatively, or in addition, strategically oriented

students are often able to manage a higher workload [10]. If setting aside time to complete the survey was perceived as increasing the workload, students with higher scores on the strategic approach may have been better prepared, and thus more inclined, to respond to it consistently over the three time points. Conversely, surface learners tend to operate from a fear of failure, often evidencing overwhelm with work and expectations, and are often inclined to spend little effort beyond what they believe is necessary to pass exams [37]. Therefore, higher dropout rates among students with higher scores on surface learning might have been expected if the addition of three surveys compounded these feelings. Strategic approach behaviors have been associated with desirable states and outcomes, such as higher self-efficacy [13] and better academic results [5, 38], while surface approach behaviors have been linked with poorer self-efficacy and poorer mental health [13], as well as poorer academic outcomes [39]. Thus, the results indicate that consistent study participation may be somewhat more commonplace among higher functioning, academically oriented students with productive study approaches. However, this needs to be further researched.

In this study, variations in the students' scores on the learning environment variables did not significantly co-vary with consistent participation in the research study. While aspects of the learning environment may lead to increased engagement [35] and satisfaction with the education program [40], they appear to be irrelevant for the retention or drop-out from a research study conducted during the course of study. While there is some evidence that females are more likely to remain engaged in longitudinal studies [33], none of the sociodemographic variables was associated with consistent participation across the three time points in this study.

Notably, participation in studies is on the decline over all, possibly due to the volume of research being done, and the amount and frequency with which people are being asked to contribute [33]. This could be a factor impacting the response rates of this study. Moreover,

there is evidence that the higher education may be becoming a more commercialized process, where students are viewed as consumers, with their feedback and experiences being routinely collected and used for course improvement, as well as for recruitment purposes [41]. This too, may have impacted students' willingness to participate in repeated data collection.

Study strengths and limitations

This research was carried out at all (six) academic institutions offering occupational therapy education in Norway, thus providing a fairly comprehensive perspective of Norwegian occupational therapy students. Nonetheless, the results require careful interpretation, as the study sample is rather small, restricted to undergraduate students in one country, and the characteristics of students opting out at all time points remain unknown. The results of the study may therefore not be fully applicable to the general occupational therapy student population. The study did not capture reasons for dropping out, nor was information obtained about the workload or other environmental factors that might explain the change in participation between the three time points.

Conclusion

The study found that rates of student participation in the study fluctuated across time, however 86 % of the eligible students participated in at least one of the three measurement times. Forty-nine percent completed the questionnaires at all time points, demonstrating that a relatively high dropout rate may be expected in a longitudinal observational study such as this. Students with higher scores on strategic approach, and lower scores on surface approach, were more likely to complete the questionnaires at each time point. There was no evidence that sociodemographic variables, features of the learning environment or student academic performance were related to consistent participation**.** These findings imply that while

substantial dropout is expected, dropout was relatively evenly distributed across sample subgroups. Thus, dropout appears not to introduce substantial sample bias in longitudinal analyses, as completers were similar to dropouts in most respects. However, students participating at all time points seem to be somewhat more well-organized and academically oriented, compared to students who did not participate at all time points. These findings may be of value for researchers and educators who are planning longitudinal studies, be they observational or experimental, among occupational therapy students.

Contributions

AG, SGJ, TC, LS, GM, TM, and TB collected the data. TB analyzed the data. ED and TB drafted the manuscript. AG, SGJ, TC, LS, GM, and TM provided scientific input and critical revisions to the different manuscript drafts. All authors approved the final version to be published and agreed to be accountable for all aspects of this work.

Competing interests

None declared.

Availability of data and materials

The dataset analyzed for this study will be available from Inland Norway University of Applied Sciences on request after the completion of the study. E-mail tore.bonsaksen@inn.no

Acknowledgements

The authors would like to thank the students who volunteered to take part in this study. In addition, we thank Kjersti Velde Helgøy (VID Specialized University, Sandnes, Norway), Vår Mathisen and Lene A. Åsli (UiT – the Arctic University of Norway, Tromsø, Norway), and Adrian W. Gran (Western Norway University of Applied Sciences, Bergen, Norway), who contributed to the data collection for this study.

References

1. Webber KL, Krylow RB, Zhang Q. Does involvement really matter? Indicators of college student success and satisfaction. J Coll Stud Dev. 2013;54(6):591-611.
2. Fredricks, JA. Academic engagement. In: Wright, JD, Ed, International Encyclopedia of the Social & Behavioral Sciences. 2nd ed. 2015, Vol. 1, San Diego: Elsevier; p. 31–36.
3. Muenks K, Wigfield A, Yang JS, O'Neal CR. How true is grit? Assessing its relations to high school and college students' personality characteristics, self-regulation, engagement, and achievement. J Educ Psychol. 2017;109(5):599.
4. Mahlberg J. Formative self-assessment college classes improves self-regulation and retention in first/second year community college students. Comm Coll J Res Pract. 2015;39(8):772-83.
5. Bonsaksen T, Brown T, Lim HB, Fong K. Approaches to studying predict academic performance in undergraduate occupational therapy students: a cross-cultural study. BMC Med Educ. 2017;17(1):1-9.
6. Wang MT, Eccles JS. School context, achievement motivation, and academic engagement: A longitudinal study of school engagement using a multidimensional perspective. Learn Instr. 2013;28:12-23.
7. Skinner E, Furrer C, Marchand G, Kindermann T. Engagement and disaffection in the classroom: Part of a larger motivational dynamic? J Educ Psychol. 2008;100(4):765.
8. Page J, Meehan-Andrews T, Weerakkody N, Hughes DL, Rathner JA. Student perceptions and learning outcomes of blended learning in a massive first-year core physiology for allied health subjects. Adv Physiol Educ. 2017;41(1):44-55.
9. Marton F, Säljö R. On qualitative differences in learning: I—Outcome and process. Br J Educ Psychol. 1976;46(1):4-11.

10. Entwistle N. Student learning and academic understanding: a research perspective with implications for teaching. Academic Press; 2018.
11. Tuononen T, Parpala A, Mattsson M, Lindblom-Ylänne S. Work experience in relation to study pace and thesis grade: investigating the mediating role of student learning. Higher Educ. 2016;72(1):41-58.
12. Entwistle N, McCune V. The conceptual bases of study strategy inventories. Educ Psychol Rev. 2004;16(4):325-45.
13. DaLomba E, Mansur S, Bonsaksen T, Greer MJ. Exploring graduate occupational and physical therapy students' approaches to studying, self-efficacy, and positive mental health. BMC Med Educ. 2021;21(1):1-8.
14. Portney LG. Foundations of clinical research: applications to evidence-based practice. FA Davis; 2020.
15. Gustavson K, von Soest T, Karevold E, Røysamb E. Attrition and generalizability in longitudinal studies: findings from a 15-year population-based study and a Monte Carlo simulation study. BMC Public Health. 2012;12(1).
16. Biering K, Hjollund NH, Frydenberg M. Using multiple imputation to deal with missing data and attrition in longitudinal studies with repeated measures of patient-reported outcomes. Clin Epidemiol. 2015;7:91.
17. Teague S, Youssef GJ, Macdonald JA, Sciberras E, Shatte A, Fuller-Tyszkiewicz M, Greenwood C, McIntosh J, Olsson CA, Hutchinson D. Retention strategies in longitudinal cohort studies: a systematic review and meta-analysis. BMC Med Res Methodol. 2018;18(1):1-22.
18. Carson R, Mennenga H. Team-Based Learning and the Team-Based Learning Student Assessment Instrument (TBL–SAI): A Longitudinal Study of Master of Occupational

Therapy Students' Changing Perceptions. Am J Occup Ther. 2019;73(4):7304205010p1-7.

19. Gribble N, Ladyshewsky RK, Parsons R. The impact of clinical placements on the emotional intelligence of occupational therapy, physiotherapy, speech pathology, and business students: a longitudinal study. BMC Med Educ. 2019;19(1).
20. Berger LK, Begun AL, Otto- Salaj LL. Participant recruitment in intervention research: scientific integrity and cost- effective strategies. Int J Soc Res Methodol. 2009;1(12):79-92.
21. Ramsden P. A performance indicator of teaching quality in higher education: The Course Experience Questionnaire. Stud Higher Educ. 1991;16(2):129-50.
22. Byrne M, Flood B. Assessing the teaching quality of accounting programmes: An evaluation of the Course Experience Questionnaire. Assess Eval Higher Educ. 2003;28(2):135-45.
23. Ginns P, Prosser M, Barrie S. Students' perceptions of teaching quality in higher education: The perspective of currently enrolled students. Stud Higher Educ. 2007;32(5):603-15.
24. Lizzio A, Wilson K, Simons R. University students' perceptions of the learning environment and academic outcomes: implications for theory and practice. Stud Higher Educ. 20021;27(1):27-52.
25. Pettersen RC. Students' experience with and evaluation of teaching and the learning environment: Presentation of the Course Experience Questionnaire (CEQ) and validation of three Norwegian versions. Halden: Østfold University College, 2007.
26. Bonsaksen T, Småstuen MC, Thørrisen MM, Fong K, Lim HB, Brown T. Factor analysis of the Approaches and Study Skills Inventory for Students in a cross- cultural

occupational therapy undergraduate student sample. Austral Occup Ther J. 2019;66(1):33-43.

27. Tait H, Entwistle NJ, McCune V. ASSIST: A reconceptualisation of the approaches to studying inventory. Improving students as learners. 1998:262-71.
28. Diseth Å. Validation of a Norwegian version of the Approaches and Study Skills Inventory for Students (ASSIST): Application of structural equation modelling. Scand J Educ Res. 2001;45(4):381-94.
29. Gramstad A, Åsli LA, Johnson SG, Magne TA, Carstensen T, Mørk G, Stigen L, Bonsaksen T. Approaches to studying: A cross-sectional comparison of occupational therapy students in six education programs in Norway. Open J Occup Ther, 2020; 8(2).
30. The Norwegian Association of Higher Education Institutions. The grading system – general, qualitative descriptions; Aug 2004; revised July 23, 2011. Available from: https://www.uhr.no/_f/p1/i4bfb251a-5e7c-4e34-916b-85478c61a800/karaktersystemet_generelle_kvalitative_beskrivelser.pdf
31. Long K, McEvoy M, Lewis L, Wiles L, Williams M, Olds T. Entry-level evidenced-based practice training in physiotherapy students: Does it change knowledge, attitudes, and behaviours? A longitudinal study. Internet J Allied Health Sci Pract. 2011;9(3):5.
32. Wong RL, Fahs DB, Talwalkar JS, Colson ER, Desai MM, Kayingo G, Balanda M, Luczak AG, Rosenthal MS. A longitudinal study of health professional students' attitudes towards interprofessional education at an American university. J Interprof Care. 2016;30(2):191-200.
33. Galea S, Tracy M. Participation rates in epidemiologic studies. Ann Epidemiol 2007;17(9):643-53.

34. DaLomba EJ, Kavanaugh E, Manbeck K, O'Neill S, Soldevilla K, Watson M, McLaughlin E. The Use of an Embedded Librarian to Enhance Student Information Literacy Skills: A Pilot Study. J Occup Ther Educ. 2020;4(2):4.
35. Fredricks JA. Engagement in school and out-of-school contexts: A multidimensional view of engagement. Theory Pract. 2011;50(4):327-35.
36. Mørk G, Magne TA, Carstensen T, Stigen L, Åsli LA, Gramstad A, Johnson SG, Bonsaksen T. Associations between learning environment variables and students' approaches to studying: a cross-sectional study. BMC Med Educ. 2020;20:1-8.
37. Richardson M, Abraham C, Bond R. Psychological correlates of university students' academic performance: a systematic review and meta-analysis. Psychol Bull. 2012;138(2):353.
38. Trigwell K, Ellis RA, Han F. Relations between students' approaches to learning, experienced emotions and outcomes of learning. Stud Higher Educ. 2012;37(7):811-24.
39. Liew SC, Sidhu J, Barua A. The relationship between learning preferences (styles and approaches) and learning outcomes among pre-clinical undergraduate medical students. BMC Med Educ. 2015;15(1):1-7.
40. Thygesen H, Gramstad A, Åsli LA, Stigen L, Magne TA, Carstensen T, Bonsaksen T. Associations between learning environment factors and student satisfaction among occupational therapy students. Irish J Occup Ther. 2020; 48(2), 91-100.
41. Woodall T, Hiller A, Resnick S. Making sense of higher education: Students as consumers and the value of the university experience. Stud Higher Educ. 2014;39(1):48-67.
